# Supplementary material for: Identification of photoperiod-induced specific miRNAs in the adrenal glands of Sunite sheep (Ovis aries)
Source: Front Vet Sci. 2022 Jul 22;9:888207. doi: 10.3389/fvets.2022.888207 (PMC9354845; doi:10.3389/fvets.2022.888207)
Supplement: Supplementary file 1 [file Data_Sheet_1.ZIP › Supplementary file/miRDeep2 file.zip!/miRDeep2 file/Know_Structure/LP42A3/oar-mir-30d.pdf]

miRBase precursor : oar-mir-30d
Total read count : 206304
oar-miR-30d read count : 205513
remaining reads : 791

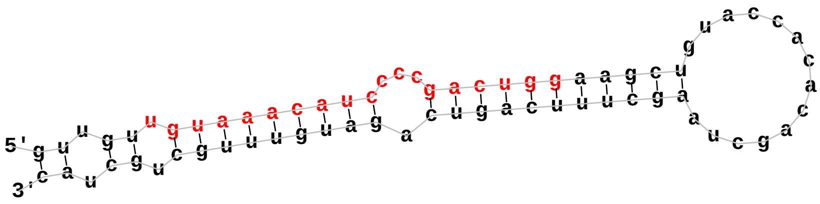

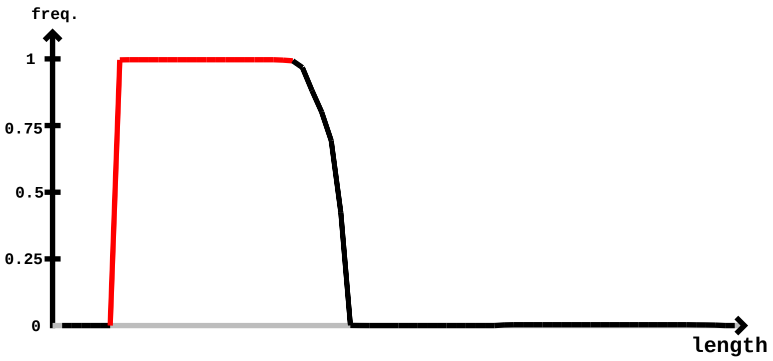


```
      oar-miR-30d
5'- guuguuguaaacauccccgacuggaagcuguaccacacagcuaagcuuucagucagauguuugcugcuac   -3'    exp
    ...((.(((((((((...(((((((((((..............))))))))))).))))))))).))...    reads      mm        sample
    ....uuguaaacauccccgacuggaagc..........................................    1          0         seq
    ....uuguaaacauccccgacuggaagcu.........................................    2          0         seq
    .....uguaaacaucUccgacu................................................    1          1         seq
    .....uguaaacauccccgacu................................................    298        0         seq
    .....uguaaacaucccUgacu................................................    5          1         seq
    .....uguaaacauccccgGcu................................................    2          1         seq
    .....uguaaacauccccgacug...............................................    569        0         seq
    .....uguaaacauccccgacugg..............................................    5123       0         seq
    .....uguaaacauccccgacugga.............................................    17804      0         seq
    .....uguaaacauccccgacuggaa............................................    16532      0         seq
    .....uguaaacauccccgacuggaag...........................................    22165      0         seq
    .....uguaaacauccccgacuggaagc..........................................    55642      0         seq
    .....uguaaacauccccgacuggaagcu.........................................    87251      0         seq
    .....uguaaacauccccgacuggaagcug........................................    87         0         seq
    .....uguaaacauccccgacuggaagcugu.......................................    39         0         seq
    .....uguaaacauccccgacuggaagcugua......................................    1          0         seq
    .....uguaaacauccccgacuggaagcuguaccaca.................................    1          0         seq
    .....uguaaacauccccgacuggaagcuguaccacacagcuaag.........................    1          0         seq
    ......guaaacauccccgacugga.............................................    9          0         seq
    ......guaaacauccccgacuggaa............................................    5          0         seq
    ......guaaacauccccgacuggaag...........................................    15         0         seq
    ......guaaacauccccgacuggaagc..........................................    29         0         seq
    ......guaaacauccccgacuggaagcu.........................................    53         0         seq
    ......guaaacauccccgacuggaagcug........................................    2          0         seq
    .......uaaacauccccgacuggaagc..........................................    2          0         seq
    .......uaaacauccccgacuggaagcu.........................................    1          0         seq
    ........aaacauccccgacuggaagc..........................................    1          0         seq
    ..........acauccccgacuggaagc..........................................    1          0         seq
    ..........acauccccgacuggaagcu.........................................    1          0         seq
    ...........cauccccgacuggaagc..........................................    1          0         seq
    ...........................................agcuuucagucGgauguuu........    1          1         seq
    .............................................cuuucagucagauguuugc......    6          0         seq
    .............................................cuuucagucagauguuugcu.....    79         0         seq
    .............................................cuuucagucagauguuugcug....    52         0         seq
    .............................................cuuucagucagauguuugcugc...    134        0         seq
```

oar-miR-30d

```
guuguuguaaacauccccgacuggaagcuguaccacacagcuaagcuuucagucagauguuugcugcuac
.............................................cuuucagucagauguuugcugcu..    216    0    seq
.............................................cuuucagucagauguuugcugcua.    3      0    seq
..............................................uuucagucagauguuugcu.....    7      0    seq
..............................................uuucagucagauguuugcug....    11     0    seq
..............................................uuucagucagauguuugcugc...    26     0    seq
..............................................uuucagucagauguuugcugcu..    121    0    seq
..............................................uuucagucagauguuugcugcua.    2      0    seq
..............................................uuucagucagauguuugcugcuac    2      0    seq
```
